# Perspective: What a Difference a Disaster Makes: The Telehealth Revolution in the Age of COVID-19 Pandemic

**DOI:** 10.1177/1062860620933587

**Published:** 2020-06-11

**Authors:** John R. Maese, Donna Seminara, Zeel Shah, Anita Szerszen

**Affiliations:** 1 Northwell Staten Island University Hospital, Staten Island, NY

**Keywords:** telehealth, telemedicine, COVID-19 pandemic, technology

## Abstract

Despite the existence of telemedicine since the late 1950s and early 1960s, it took a pandemic to bring this technology mainstream. The critical urgency of the pandemic drove an auspicious alignment of policy, economics, and technology to facilitate the widespread implementation of telehealth. It is imperative that this synchronicity be maintained in the post-COVID era in order to optimize our health care system to be ready for the next threat to the health of the United States.

Necessity is the mother of invention. The COVID-19 pandemic has forced medicine to reevaluate current clinical practice. The State of New York defines telemedicine as a 2-way electronic audiovisual communication to deliver clinical health care services to a patient at an originating site by a telehealth provider located at a distant site. The totality of the communication of information exchanged between the physician or other qualified health care practitioner and the patient during the course of the synchronous telemedicine service must be of an amount and nature that would be sufficient to meet the key components and/or requirements of the same service when rendered via face-to-face interaction.^1^ Telemedicine has existed since the late 1950s and early 1960s^2^; however, it was not financially viable to have widespread adoption until this century. Mainstream adoption of telemedicine has been limited by policy, technology, and economics. Policies regarding licensure, credentialing, and malpractice limited physician adoption. Telemedicine technology was expensive and slow to facilitate a meaningful interaction between the patient and the provider. Technological limitations included the hardware, software, and internet access. Economic policies limited telemedicine as an effective clinical option except in underserved areas or in an emergency. Insurance companies placed limitations on who could offer this technology. Large telemedicine service providers were contracted by insurance companies to offer these services instead of the patient’s own primary care physician. Psychiatry^3^ and prison^4^ medicine also have been able to successfully implement telemedicine because of the discrepancy in the available supply of providers and the need.

As technology evolved and electronic medical records became the norm, it allowed for integration with other technologies such as smartphones, and this made telemedicine a popular option among younger patients. Despite a growing desire to have widespread adoption of telemedicine, limitations continued to allow only larger practices and health systems to incorporate this service. We have seen technologies such as the iPod and the iPhone trigger a paradigm shift and dramatically change the landscape of our daily life. Adoption of innovation and technology on such a broad scale requires a catalyst and the current pandemic may be the disruption we needed to make a lasting change.

The COVID-19 pandemic has pushed telehealth to the forefront in a way that 30 years of physician advocacy could not. The pandemic forced the alignment of the policy, technology, and economics of telemedicine. Fear of the contagion changed patients’ attitudes toward telehealth. During the outbreak, the federal government relaxed the policies surrounding telemedicine so that physicians could continue to treat their patients while mitigating the spread of disease. The most groundbreaking change was the easing of privacy regulation.

The federal government stated that during the pandemic,

Covered health care providers will not be subject to penalties for violations of the HIPAA Privacy, Security, and Breach Notification Rules that occur in the good faith provision of telehealth during the COVID-19 nationwide public health emergency. This Notification does not affect the application of the HIPAA Rules to other areas of health care outside of telehealth during the emergency.^5^

The implications of this ruling sent shockwaves through the health care industry. This was a seismic change because doctors and patients could finally use existing technology without fear. The federal government allowed the use of “non–public facing” remote communication products to provide telemedicine services during this crisis. Non–public facing remote communication products, as a default setting, allow only the intended parties to participate in the communication. These products include popular applications such as Apple FaceTime, WhatsApp video chat, Zoom, and Skype. This permitted doctors and patients to use ubiquitous familiar technology such as FaceTime, which previously would not have been possible because FaceTime is not HIPAA-compliant. FaceTime is appropriately encrypted but Apple did not sign a business associates’ agreement and FaceTime normally could not be used. The modification of the rules allowed for telemedicine to take place in a variety of locations. The federal government relaxed limitations on the services that could be provided by telehealth. The federal government stated that

all services that a covered health care provider, in their professional judgement, believes can be provided through telehealth in the given circumstances of the current emergency are covered by this Notification. This includes diagnosis or treatment of COVID-19 related conditions, etc.^5^

This ruling expands the number of patients that could been seen via telehealth and drove significant reimbursement reform. Additional regulations by the federal and state governments to pay telemedicine visits at the same rates as an in-office visit have encouraged widespread adoption. A long-standing taboo in telehealth has been that the physician has to be licensed in the same state as the patient. The rationale for these antiquated rules has to do with licensing fees to states, jurisdiction over misconduct, and the location of a malpractice action. The easing of malpractice rules and licensing fees reduced significant barriers to adoption of telemedicine.

Alignment of these forces is demonstrated by a nonscientific SurveyMonkey poll we conducted with members of the local County Medical Society. We polled the members of the Society regarding the use of telemedicine pre and post COVID-19. Responses indicated that 15% of members were using telemedicine prior to the pandemic. After the pandemic started, 89% of members responded that they were offering telemedicine services. This was not a scientific study; however, the results do illustrate the power that synergy between policy, technology, and economics has in disrupting the marketplace and changing behavior acutely.

Several changes need to be addressed in order to effectively integrate telemedicine into the public health response to COVID-19. These include a need for a regulatory framework to authorize, integrate, and reimburse telemedicine in the delivery of care for all patients, especially in emergency situations.^6^ The Centers for Medicare & Medicaid Services took a step toward facilitating this with an 1135 Waiver to expand telehealth coverage for all Medicare patients during the COVID-19 pandemic. Many of the restrictions, namely a lack of reimbursement, licensing restrictions, and HIPAA compliance, which previously were roadblocks to telehealth integration, have been removed to promote “good faith use of telehealth.”^7^ Thoughtful implementation of telemedicine now may allow for sustainable and scalable practice beyond the current crisis while maintaining high standards for patient privacy and data security.^8^

Physicians need to have a dialogue with legislative leaders to find a balance between the old regulations and the emergency regulations. Many practices will not survive in the post COVID-19 era without relaxed regulation that will allow for routine use of telemedicine. Locally, physicians have seen a dramatic reduction in total visits and in-person office visits.^9^ In a survey of Medical Society physicians, 90% decreased their hours in the office because patients would not come in to be seen. A majority of those physicians decreased their hours by as much as 12 hours per week. Telemedicine will be the key to practice survival. If we want physicians to embrace technology, to transform and innovate the practice of medicine, we need to keep them safe from prosecutory harm, regulate in a sensible good faith manner, and continue to reimburse practices for telephone and telehealth visits at the same rate as an office visit. If reimbursement policy does not reflect the new paradigm, practices will close and many private practice physicians will opt for early retirement. This will further exacerbate the current physician shortage. Access to care will suffer dramatically for patients. COVID-19 has demonstrated the necessity of a public, private, and physician alignment to keep our health care system intact to treat any threats to the health of the United States.
